# Thank You to Our GeoHealth 2024 Reviewers

**DOI:** 10.1029/2025GH001412

**Published:** 2025-03-18

**Authors:** Thanh H. Nguyen

**Affiliations:** ^1^ University of Illinois at Urbana Champaign Urbana IL USA

**Keywords:** editorial, peer review

## Abstract

Peer‐review is the foundation and the safeguard of scientific research. Without the dedication of our reviewers, the journal would not have been successful. In 2024, 231 reviewers completed the review for 238 manuscripts submitted to GeoHealth. Our reviewers are from all continents except Antarctica. Besides reviewers from North America, China, Europe, and China, we started to have reviewers from India, Latin America, and Africa. GeoHealth editorial board is committed to expanding the readership, authorship, and reviewership to other countries. If you have already reviewed for us, no matter where or who you are, we hope you and your colleagues will consider GeoHeatlh a home for your work. Below is the list of reviewers who completed more than two reviews (*noted in italics*) or have outstanding quality reviews. Two of our reviewers have been nominated for AGU Best Reviewers awards (*noted with an asterisk).

## Key Points

- The editors thank the 2024 peer reviewers

*Akanda, Ali Shafqat*

Alwadi, Yazan

*Andrew Essien, Elizabeth E.*

*Biswas, Nishan Kumar*

*Boger, Rebecca*

*Bozigar, Mattew*

*Brimicome, Chloe*

Burger, Moritz

*Carrión, Daniel*

*Carruthers, David*

*Chen, Kai*

*Chipman, Jonathan W.*

Chu, Lingzhi

Chung, Maya V.

*Cleland, Stephanie E.*

*Crooks, James L.*

Elbanna, Ahmed

*Engel‐Di Mauro, Salvatore*

Farguell Caus, Angel

*Gangwar, Mayank*

*Gani, Shahzad*

*Gibson, Seth*

Gilmore, Troy E.

*Goncalves, Alexandre*

*Grover, Elise*

*Guimarães, Ricardo J.*

*Harp, Ryan D.*

*He, Cheng*

*He, Huan*

*He, Joe*

Head, Jennifer

*Heaney, Alexandra*

Heft‐Neal, Sam

*Holcomb, Karen M.*

Hu, Kai

*Huang, Keyong*

*Hyland, Carly*

*Jamal, Yusuf*

*Johnson, Daniel*

*Joseph, Naveen*

Kaufeld, Kimberly

Kephart, Josiah

*Kong, Qinqin*

Koolik, Libby

Kumar, Mukesh

*Le, Phong V.*

Lee, Jangho

Leonard, Kelsey

*Li, Lingli*

*Limaye, Vijay S.*

*Liu, Fangchao*

*Liu, Fengyun*

*Ma, Xuying*

*Ma, Yiqun*

Magers, Bailey*

*Masoumi, Zohreh*

*Meng, Wenjun*

*Nawaz, Muhammad O.*

Nawaz, Omar

Ng, Chris Fook Sheng*

*O.O, Oketayo*

Ogneva‐Himmelberger, Yelena

Ojeda, Ann

*Pan, Hai‐Feng*

Pascual, Mercedes

*Puvvula, Jagadeesh*

*Qiu, Minghao*

*Qiu, Ning*

Richmond‐Bryant, Jennifer

*Ryan, Sophia C.*

*Schollaert Uz, Stephanie*

Shen, Dixin

*Shen, Huizhong*

Stiles, Pascale

*Sun, Zhaobin*

Swope, Carolyn

Taiba, Jabeen

Tessum, Christopher

*Tong, Daniel*

*Usmani, Moiz*

*Vohra, Karn*

*Wang, Dashan*

Wang, Pin

*Wang, Xiaolin*

*Wang, Yuxuan*

*Wang, Yuzhou*

*Xiaio, Qian*

*Xiong, Ying*

*Xue, Tao*

*Yuan, Jiacan*

*Yuan, Lei*

Zaitchik, Benjamin F.

*Zhao, Dajun*

*Zhao, Lining*

*Zhu, Qingyang*

